# Fast-track treatment initiation counselling in South Africa: A cost-outcomes analysis

**DOI:** 10.1371/journal.pone.0248551

**Published:** 2021-03-18

**Authors:** Bruce A. Larson, Sophie J. S. Pascoe, Amy Huber, Lawrence C. Long, Joshua Murphy, Jacqui Miot, Nicole Fraser-Hurt, Matthew P. Fox, Sydney Rosen

**Affiliations:** 1 Department of Global Health, Boston University School of Public Health, Boston, Massachusetts, United States of America; 2 Faculty of Health Sciences, Health Economics and Epidemiology Research Office, Department of Internal Medicine, School of Clinical Medicine, University of the Witwatersrand, Johannesburg, South Africa; 3 The World Bank, Washington, DC, United States of America; 4 Department of Epidemiology, Boston University School of Public Health, Boston, Massachusetts, United States of America; University of South Carolina College of Pharmacy, UNITED STATES

## Abstract

**Introduction:**

In 2016, under its new National Adherence Guidelines (AGL), South Africa formalized an existing model of fast-track HIV treatment initiation counselling (FTIC). Rollout of the AGL included an evaluation study at 24 clinics, with staggered AGL implementation. Using routinely collected data extracted as part of the evaluation study, we estimated and compared the costs of HIV care and treatment from the provider’s perspective at the 12 clinics implementing the new, formalized model (AGL-FTIC) to costs at the 12 clinics continuing to implement some earlier, less formalized, model that likely varied across clinics (denoted here as early-FTIC).

**Methods:**

This was a cost-outcome analysis using standard methods and a composite outcome defined as initiated antiretroviral therapy (ART) within 30 days of treatment eligibility and retained in care at 9 months. Using patient-level, bottom-up resource-utilization data and local unit costs, we estimated patient-level costs of care and treatment in 2017 U.S. dollars over the 9-month evaluation follow-up period for the two models of care. Resource use and costs, disaggregated by antiretroviral medications, laboratory tests, and clinic visits, are reported by model of care and stratified by the composite outcome.

**Results:**

A total of 350/343 patients in the early-FTIC/AGL-FTIC models of care are included in this analysis. Mean/median costs were similar for both models of care ($135/$153 for early-FTIC, $130/$151 for AGL-FTIC). For the subset achieving the composite outcome, resource use and therefore mean/median costs were similar but slightly higher, reflecting care consistent with treatment guidelines ($163/$166 for early-FTIC, $168/$170 for AGL-FTIC). Not surprisingly, costs for patients not achieving the composite outcome were substantially less, mainly because they only had two or fewer follow-up visits and, therefore, received substantially less ART than patients who achieved the composite outcome.

**Conclusion:**

The 2016 adherence guidelines clarified expectations for the content and timing of adherence counseling sessions in relation to ART initiation. Because clinics were already initiating patients on ART quickly by 2016, little room existed for the new model of fast-track initiation counseling to reduce the number of pre-ART clinic visits at the study sites and therefore to reduce costs of care and treatment.

**Trial registration:**

Clinical Trial Number: NCT02536768.

## Introduction

South Africa’s National Department of Health (NDOH) launched the National Adherence Guidelines for Chronic Diseases in 2015 [1]. The guidelines, which were essentially a set of adherence-related interventions, included “fast-track treatment initiation counselling” (FTIC) [2]. FTIC was designed to standardize HIV treatment education, counselling, and support provided to patients starting antiretroviral therapy (ART) without delaying treatment initiation. The goal of FTIC was to cut down on losses between known eligibility for ART and ART initiation by reducing the number of clinic visits prior to initiation and re-structuring the counseling and education program during clinic visits, potentially altering the clinic’s resource utilization and thus costs of service delivery per patient.

Prior to national roll out of the guidelines, the NDOH and its partners identified 24 clinics where guideline implementation would be staggered to facilitate a cluster-randomized study to evaluate impacts of the adherence interventions on various treatment outcomes. Patients were enrolled in this Adherence Guidelines Evaluation (AGL) study between January and December 2016, with follow up through 2017 (see the study protocol for further details regarding clinic locations and randomization procedures [2]). Results from the AGL study showed a modest (6%) increase in the proportion of patients initiated within 30 days but a modest decrease in the proportion retained over the original 9 months main follow up period (a 7% decrease), which largely dissipated by 12 months [3].

By January 2016, however, when enrollment began, a large proportion of HIV-positive individuals were already eligible to start ART immediately upon diagnosis with HIV, based on 2015 treatment guidelines [4]. All patients became eligible for ART upon diagnosis in September 2016, when South Africa adopted universal treatment [5].

Expectations for HIV-related counseling before and after treatment initiation were also evolving prior to the roll out of the new adherence guidelines. Until 2012, patients in South Africa were expected to complete three counseling sessions prior to ART initiation, typically at three separate “pre-ART” clinic visits. In 2012, however, the NDOH released a recommendation to “fast-track” certain groups of patients onto treatment (initiate within 7 days of eligibility), and, in response, some clinics initiated patients with minimal adherence counseling, while others continued to require multiple sessions before initiation [1]. By 2015, guidelines stated that ART should be started as soon as a patient was ready, generally within 14 days of eligibility, and that certain patients should be fast-tracked for initiation (within 7 days) [4]. The 2015 guidelines did not specify the timing of adherence counseling relative to ART initiation, but they did state that such counseling should begin on the day of HIV diagnosis, be provided monthly for the next 3 months, and then be offered quarterly. As of result of this gradual evolution of treatment initiation and counseling guidelines, public clinics in South Africa, including the 24 AGL evaluation study clinics, were already implementing some form of “faster-track” initiation that incorporated adherence counseling when the AGL were released. They did not, however, have consistent expectations on the timing and content of counseling sessions relative to ART initiation [6]. While data are not available to document these earlier practices in detail, it is likely that practices across clinics, and practices for patients within the same clinics, varied widely.

With the newer, formalized FTIC model, the AGL provided explicit guidance on the timing and content of adherence counseling sessions relative to ART initiation. The guidelines called for patients to receive four adherence counseling sessions: one on the day of ART eligibility; one session seven days later (which would have been 7 days after HIV diagnosis and the day of initiation for some patients; others who received same-day initiation might already have reached day seven on ART); and then two additional counseling sessions at subsequent clinic visits (at one- and two-months on ART). The overall goal of this new FTIC model was to standardize adherence counseling (timing and content) as part of the initiation process and reduce barriers to ART initiation, with the goal of increasing adherence and retention. A simple, written adherence plan is envisioned to be completed with the patient during these sessions, and a two-page sample plan is included in the AGL appendix.

To complement the effectiveness results from the AGL evaluation study, the purpose of this analysis is to estimate the costs of HIV care and treatment from the provider’s perspective for patients enrolled in the AGL evaluation study from the day of treatment eligibility through a 9-month follow-up period (the original primary period for the AGL study [3]) at the 12 AGL evaluation sites assigned to early rollout of guidelines and the 12 comparison sites assigned to delayed roll out of the guidelines.

## Methods

### Overview

A basic cost-outcomes analysis, applying standard methods used in related studies of the cost of HIV care and treatment [7–9], is used to estimate patient-level costs of care and treatment over a 9-month study follow up period (the original “long-term” follow-up period for the AGL evaluation study) at 24 primary healthcare clinics in four provinces of South Africa (Gauteng, KwaZulu‐Natal, Limpopo, and North West). The AGL study selected three pairs of high‐volume HIV treatment facilities (>1000 ART patients) from a single district in each province (24 total) [3]. Pairs of sites were matched on viral suppression, setting (rural/urban/formal/informal) and location (geographic proximity). Within each pair, one facility was randomly allocated to implement the adherence guidelines interventions and one randomly selected to serve as a comparison (standard of care), giving us a cluster‐randomized evaluation design (and see [3] for additional details). The 12 comparison clinics, with delayed assignment to begin implementation of the guidelines, are called the “early-FTIC” model of care, while acknowledging the possibility of wide variation in actual practices across the clinics. The 12 intervention clinics, where the AGL guidelines were implemented during the study period, are considered the “AGL-FTIC” model of care.

For patient costs over the specific follow-up period for the analysis, we calculated the quantities of specific resources used for each patient, and then based on a unit cost for each resource, we calculated costs over the time period for each patient (resource quantities times unit cost). We address each topic below in more detail.

The full AGL evaluation study was approved by the Human Research Ethics Committee (HREC) of the University of the Witwatersrand and the Boston University Institutional Review Board (IRB). Both approved use of routine clinic data for the evaluation (including the evaluation of costs) and a waiver of patient consent. The trial is registered at clinicaltrials.gov (NCT02536768).

### Time period for patient follow up

**Fig 1:**
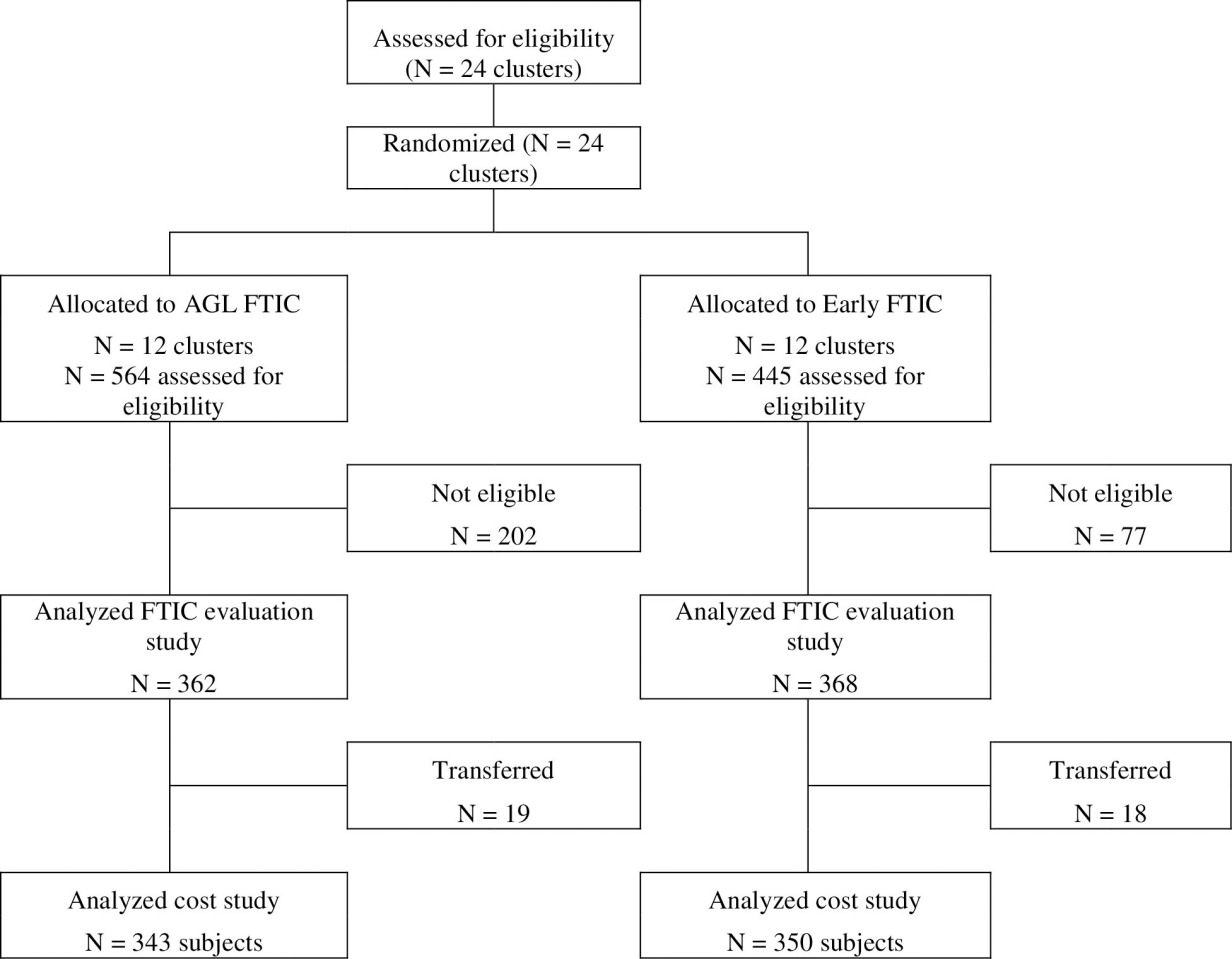
Flow diagram for fast track initiation counseling cohort creation and analyses.

For this analysis, the follow-up period is taken from the follow-up-start date as defined by the AGL evaluation, which for the FTIC portion of the overall study was from the day of treatment eligibility at the clinic through 270 days after the follow up start date, which coincides with the study primary endpoint of viral suppression within 9 months (see [2], Fig 1). Patients who transferred to other clinics (known transfers) are excluded from this costing analysis because they could not have a full 9 months of follow-up data.

### Resource use (input quantities)

This analysis focuses on three categories of resources used for patient care during the study follow up period: ARV medications, laboratory tests, and clinic visits. Data for each patient came from three sources: (1) a national electronic patient database (TIER.Net) which provides information on visit date, ARV regimen, number of pills collected, and laboratory tests; (2) paper-based clinic records (clinic registers, patient clinic files) which were used by AGL study staff to review patient enrolment in AGL-FTIC at the intervention sites and counselling sessions at the standard model sites; and (3) the National Health Laboratory Service (NHLS) database of all viral loads done in South Africa’s public sector.

While the AGL evaluation data are adequate to identify the category of a clinic visit (e.g., before ART initiation, the day of initiation, and post-initiation visits), study clinics typically did not record the timing and content of counselling sessions [3]. Thus, data do not actually exist to document the number and timing of adherence counseling visits received by each patient in the study clinics in either the early-FTIC or AGL-FTIC models. The implications of such missing information is considered further in the discussion section.

As noted above, patient-level data were extracted from the national electronic patient database, TIER.Net. While the AGL study team worked with facility staff on data enhancement and completion of missing data during a short period at the beginning of the study, the overall quality of the data collected at sites and entered into TIER.Net at the 24 study sites was largely beyond the control of the AGL study.

### Unit costs

All costs are reported in 2017 U.S. dollars (US$) using the 2017 average annual exchange rate (13.32 ZAR/US$) reported in the International Monetary Fund’s International Financial Statistics [10]. ***S1 File*** provides all primary information used along with additional sources as needed to estimate all unit costs used in this analysis.

For ARV medications, costs were obtained from official tender documents and converted to a daily equivalent [11]. For laboratory tests, the unit costs were obtained from the NHLS master fee schedule for 2017 [12]. The NHLS fees also include the cost of sample transport.

Unit costs for three types of visits are included in this analysis: pre-initiation visits, the initiation visit, and the post-initiation follow-up visits. All clinics included in the AGL study were Primary Health Care (PHC) clinics. The unit cost for a standard PHC clinic visit for HIV patients on treatment is from a recent paper by Larson et al [13], which is based on an earlier study by Long et al [8] and inflation adjusted to 2017 using South African consumer price inflation [14]. In addition, the cost for an ART initiation visit completed with a nurse is also based on a recent South African study [7]. For perspective, these visit costs are compared to the time-equivalent salary cost for professional nurses based on government salary scales from the Department of Public Service and Administration.

The unit cost for a pre-ART visit is somewhat less clear. The AGL evaluation reported that patient records or systems were not adequate to identify if patients received counseling at clinic visits, and as a result the “extent and content of counselling provided remains unknown and likely varies by facility and healthcare provider” [3]. In the absence of better data, the unit cost for a pre-ART initiation visit is assumed to be equal to the cost for a standard follow up clinic visit. If there are many pre-ART visits in the data, and substantial differences between the quantity of such visits by model of care, then this assumption is important in the analysis and a better estimate would be needed. However, for this analysis, the data show very few pre-ART visits for both models of care, both in absolute numbers and relative to the number of follow-up visits, so this assumption is not important for this analysis.

### Reporting results

The mean, median and interquartile range (IQR) for resource quantities (days of ARVs, number of laboratory tests, quantity of visits by type) and costs are reported for each model of care. 95% confidence intervals for mean total costs are also reported (using the *reg* command in STATA with the cluster by site option).

In the cost-outcomes literature, costs are also typically reported stratified by patients achieving or not achieving a certain outcome, such as retention in care at the end of the study follow up period (based on varying definitions) [7,8,15–19]. For this analysis of AGL-FTIC, a composite outcome (initiated and retained in care) is defined as a patient initiated on ART within 30 days of treatment eligibility (the short-term outcome in the AGL study); and was retained in care at 9 months (defined as completing a clinic visit during the 180–270 day period study follow up). If a patient satisfies both conditions, then they achieved this outcome. This composite outcome is consistent with the basic goals of FTIC, which is to initiate patients on ART promptly after known eligibility and retention after initiation. This composite outcome is also consistent with recent studies focused on both ART initiation and retention (see, e.g. [20]), and South African policy which aims to support ART initiation with limited delays and ensure patient retention.

The composite outcome used here is not exactly comparable to the final 9-month endpoint in the AGL study. The 9-month outcome envisioned in the study protocol was viral suppression “within 9 months”, which was operationalized as viral suppression for any test completed during month 2 to 9 of study follow up. We did not consider a composite outcome using the viral suppression outcome as appropriate because it was based on any test during month 2–9 of follow up. For example, with any viral suppression between month 2–9, a patient could achieve this outcome but initiate ART on day 40 of follow up (so failing to achieve the short-term outcome), have a viral load test 3 months later that is suppressed (so achieving the 9-month primary outcome), but then be lost to follow up for the remaining study period. Costs for this person over 9-months are low because they only receive perhaps three months of ART, which is clearly not the intent of the South African AGL policy.

## Results

### Analytic sample and primary outcome

Fig 1 provides a CONSORT flow diagram for the study. From the total sample of subjects analyzed in the AGL study (362 AGL FTIC; 368 Early FTIC), a total of 37 subjects transferred and were excluded from the costing analysis (18 Early FTIC; 19 AGL FTIC). The AGL evaluation concluded that the two study arms were “largely balanced on baseline characteristics” (see Table 1 in [3]). In ***S1 File***, we also show in S1 Table that patient characteristics for the costing cohorts (transfers excluded) remain largely balanced and essentially the same as the full AGL evaluation cohorts.

Table 1 summarizes the analytic sample size and the sample sizes for stratifying results by achievement of the composite outcome.

**Table 1:** Sample size and achievement of the primary outcome.

Variable	Early-FTIC (n = 350*)	AGL-FTIC (n = 343*)
Initiated ART < = 30 days: number (percent)	289 (83%)	281 (82%)
Retained**: number (percent)	268 (77%)	237 (69%)
Achieved composite outcome (initiated within 30 days and retained): number (percent)	223 (64%)	205 (60%)

* Denominator for percentages.

** Retained defined as completing a clinic visit during day 180–270 after the beginning of study follow up.

In ***S1 File***, S2 Table presents patient characteristics for both costing cohorts and stratified by composite outcome status. Patient characteristics for the subset achieving the composite outcome in each group (Early FTIC and AGL FTIC) remain largely balanced (both when comparing to the full cohort for each group and across groups for the subsets achieving the composite outcome. Characteristics for the Early-FTIC subset that did not achieve the composite outcome also remain similar to the total Early-FTIC group and subset achieving the composite outcome. The AGL-FTIC subset that did not achieve the composite outcome, however, had somewhat lower baseline CD4 cell counts than the other cohorts.

The results provided in Table 1 above are similar to the results reported previously as part of the impact evaluation study (see Table 2 and S7 Table in [3]). The results in Table 1 though, are not exactly the same as in [3] because, as explained in Fig 1, the costing sample excludes patients who transferred and the definition of “alive and in care” used in [3] is slightly different from the definition of retained in Table 1 above (based on TIER.Net processes and information available in January 2018 for all patients rather than at 9-months after study follow up for each patient).

In Table 1, note that retention for the AGL-FTIC group was lower than for the early-FTIC group, which is consistent with the AGL evaluation study results [3], but such differences disappeared by 12 months (beyond the follow up period possible for this costing analysis). In addition, in the AGL evaluation study, the intervention (AGL-FTIC) clinics were different from the comparison clinics (early FTIC), which is why a difference-in-difference approach was used to assess impact in the AGL evaluation study and the differences reported in Table 1 cannot be interpreted as impact.

### Unit costs

Table 2 lists the most important unit costs for this analysis, and all unit costs are also provided in ***S1 File*** (with references for all source materials provided). While multiple ARV regimens are available in South Africa, as reported in ***S1 File***, almost all ARVs dispensed to patients in each model of care during the study period were the main national, once-a-day, fixed-dose, first-line regimen (TDF/FTC/EFV), with a daily cost of $0.32 ($116.8 per year). Similarly, although 29 different lab tests were utilized and included in the analysis, only three tests were somewhat common (CD4, viral load, creatinine clearance, and see ***S1 File*** for details).

**Table 2:** Unit costs.

Resource	Unit	Unit cost (USD 2017)
ARVs*	1 day	0.32
**CD4 test**	1 test	4.72
**Viral load test**	1 test	24.11
**Creatinine clearance**	1 test	2.16
**Follow-up clinic visit (post initiation)**	1 visit	7.61
**Pre-ART clinic visit**	1 visit	7.61
**ART initiation visit**	1 visit	8.92

* Based on fixed-dose combination of TDF/FTC/EFV.

We recognize that visit costs vary depending on the types of staff seen at a typical visit, patient/staff volume at clinics, and/or provider time spent with a patient. For perspective, public-sector professional nurse salaries in South African are based on Grade (1, 2, or 3) and level within each grade. The median (Grade 2, level 4) annual salary in 2017 was $22,811 (see ***S1 File***). Assuming 18 working days per month (due to national holidays, annual leave, sick leave, etc.), the daily salary equivalent is $105.61. With 8 hours of work expected daily and 7 hours directly spent with patients (the one hour for time between patients included any paper work, tea, etc.), the implied salary cost per one hour of patient time is $15.09. In other words, the follow-up clinic visit in Table 2 is the equivalent of about 30 minutes of time for a professional nurse (Grade 2, level 4). On the other hand, 15 minutes of nurse time would be $3.77, but another 15–30 minutes for counseling time (with a counselor for example) and/or allowing for some non-salary costs of clinic visits would push the visit cost back towards the same cost as reported in Table 2. The implications of alternative visit costs are discussed after presenting the main results.

### Resource use and costs

Table 3 summarizes resource use and costs for each model of care.

**Table 3:** Resource use and costs of patient care (over 9 months from treatment eligibility) for each model.

	Early-FTIC (comparison sites)	AGL-FTIC (Intervention sites)	Early-FTIC	AGL-FTIC
	mean	mean	median (IQR)	median (IQR)
Days of ARVs	190.57	175.87	224 (140, 252)	210 (112, 252)
Pre-ART visits	0.98	1.01	1 (1, 1)	1 (1, 1)
Initiation visits	0.97	0.93	1 (1, 1)	1 (1, 1)
Follow-up visits	4.62	4.30	5 (3, 6)	5 (2, 6)
CD4 tests	1.02	1.04	1 (1, 1)	1 (1, 1)
Viral load tests	0.53	0.61	1 (0, 1)	1 (0, 1)
Creatinine clearance tests	0.64	0.76	1 (0, 1)	1 (0, 1)
Cost of ARVs	62.45	57.24	72.8 (45.5, 81.9)	63.76 (36.4, 81.9)
Cost of laboratory tests	20.93	24.19	28.83 (6, 30.99)	28.83 (6.88, 33.15)
Cost of visits	51.28	48.70	54.58 (39.36, 62.19)	46.97 (31.75, 62.19)
Total costs	134.66	130.13	153.13 (106.03, 172.92)	150.76 (80.48, 175.36)

Mean costs per patient were similar for the two groups, $134.66 (95% CI: $127.5, $141.9) for the early-FTIC group and $130.13 (95% CI: $119.6, $140.7) for the AGL-FTIC group, with the difference coming from fewer days of ARVs for the AGL-FTIC group than the early-FTIC group. Mean costs are lower than median costs because of the very low costs for the small number of patients who did not initiate ART or who were lost to follow up quickly after initiating ART.

Table 4 summarizes resource use and costs for each model of care for the subset achieving the composite outcome.

**Table 4:** Resource use and costs of patient care (over 9 months from treatment eligibility) for each model for the subset achieving the composite outcome.

	Early-FTIC (comparison sites)	AGL-FTIC (Intervention sites)	Early-FTIC	AGL-FTIC
	mean	mean	median (IQR)	median (IQR)
Days of ARVs	234.80	233.35	252 (224, 260)	252 (224, 260)
Pre-ART visits	0.90	0.91	1 (1, 1)	1 (1, 1)
Initiation visits	1.00	1.00	1 (1, 1)	1 (1, 1)
Follow-up visits	5.82	5.84	6 (5, 7)	6 (5, 7)
CD4 tests	1.03	1.09	1 (1, 1)	1 (1, 1)
Viral load tests	0.71	0.84	1 (0, 1)	1 (1, 1)
Creatinine clearance tests	0.72	1.00	1 (0, 1)	1 (0, 2)
Cost of ARVs	77.01	76.05	81.9 (72.8, 84.82)	81.9 (72.8, 84.82)
Cost of laboratory tests	25.61	31.18	28.83 (15.04, 31.96)	30.99 (28.83, 36.78)
Cost of visits	60.01	60.26	62.19 (46.97, 69.8)	62.19 (46.97, 69.8)
Total costs	162.63	167.50	165.82 (150.17, 178.74)	169.54 (153.23, 186.83)

Mean costs were similar for the two groups, $162.63 (95% CI: $156.82, $168.43) for the early-FTIC group and $167.50 (95% CI: $159.34, $175.65) for the AGL-FTIC group. Patients in both models who achieved this outcome had almost identical resource use and therefore costs. These patients received about 234 days of ARVs (median of 252 days), which is consistent with ART initiation within 30 days. Given that almost all ARV medications received were the standard fixed-dose combination, ARV medication costs were essentially identical for both models.

Based on the estimated means and medians for visits, patients in both models of care typically had one pre-ART visit, one initiation visit (by definition), and 6 follow-up visits over the study follow up period (270 days). Given the number of visits were identical (including pre-ART visits), visit costs were also basically identical.

Regarding laboratory tests, as part of treatment initiation, study subjects typically had one CD4 test and one creatinine clearance test. The median quantity of viral load tests was consistent with guidelines recommending viral load testing after 6 months on ART.

As the quantities of resources (ARV medications, laboratory test, visits) were almost the same for both models of care for the subset achieving the primary outcome, the cost for medications, laboratory tests, and visits were very similar as well. Results for those not achieving the composite outcome are provided in Table 5.

**Table 5:** Resource use and costs of patient care (over 9 months from treatment eligibility) for each model for the subset not achieving the composite outcome.

	Early-FTIC (comparison sites)	AGL-FTIC (Intervention sites)	Early-FTIC	AGL-FTIC
	mean	mean	median (IQR)	median (IQR)
Days of ARVs	112.90	90.48	112 (42, 168)	84 (28, 168)
Pre-ART visits	1.13	1.16	1 (1, 1)	1 (1, 1)
Initiation visits	0.92	0.83	1 (1, 1)	1 (1, 1)
Follow-up visits	2.52	2.01	2 (1, 4)	1.5 (0, 3)
CD4 tests	1.01	0.97	1 (1, 1)	1 (1, 1)
Viral load tests	0.22	0.27	0 (0, 0)	0 (0, 1)
Creatinine clearance tests	0.49	0.41	0 (0, 1)	0 (0, 1)
Cost of ARVs	36.87	29.28	36.4 (13.65, 54.6)	27.3 (9.1, 54.6)
Cost of laboratory tests	12.73	13.81	6.88 (4.72, 22.3)	6.88 (4.72, 28.83)
Cost of visits	35.96	31.53	31.75 (24.14, 46.97)	31.75 (16.53, 39.36)
Total costs	85.56	74.63	81.97 (49.22, 118.91)	65.96 (30.35, 118.07)

Not surprisingly, as shown in Table 5, patients who did not achieve the composite outcome had low costs of care: $85.56 for the early-FTIC group (95% CI: $75.77, $95.35) and $74.63 for the AGL-FTIC (95% CI: $66.47, $82.78). Costs of care for these patients are low because: (1) they initiated ART after more than 30 days (or not at all), so they had a shorter period on ART during study follow up; and/or (2) they initiated ART but did not complete a visit during the last 3 months of study follow up, so they were without ARV medications during at least an important portion of the follow up period. The mean/median days of ARVs are less for the AGL-FTIC group in Table 5, as compared to the early-FTIC group, because the AGL-FTIC group included more patients who never initiated ART during the study period. In the early-FTIC group in Table 5, 10/127 patients (about 8%) did not start ART while 23/138 (about 17%) in the AGL-FTIC did not initiate ART during the study follow-up period.

## Discussion

The AGL evaluation assessed the FTIC model of care during the early phase of rolling out the adherence guidelines, at a time when the comparison study clinics were attempting to balance competing guidance on adherence counseling but also faster ART initiation. During the study period in the non-intervention (early-FTIC) clinics, counselling as part of the standard care was guided by the National Consolidated Guidelines for PMTCT and the management of HIV in children, adolescents and adults but was not always standardized across sites [1]. At the same time, other guidelines stated that ART should be started as soon as a patient was ready, generally within 14 days of eligibility, and that certain patients should be fast-tracked for initiation (within 7 days) [4]. One goal of the new AGL-FTIC guidelines were to standardize adherence counseling as part of a faster ART-initiation process. Given that standard care before the implementation of the national adherence guidelines recommended multiple visits before ART initiation [3], a reasonable expectation was that the cost of pre-ART care would fall, mainly from fewer visits. The results presented here (see Tables 3 and 4) show, however, that cost reductions for pre-ART care had already been achieved at the early-FTIC clinics because they had already evolved to one pre-ART visit by the time the guidelines were rolled out to the AGL study intervention clinics.

Average costs over the 9-month follow up period were very similar for both study groups (early-FTIC/AGL-FTIC = $134/130 from Table 3, and $162/167 for the subset achieving the composite outcome). Costs for pre-ART care were a minor share of total costs; patients in either study group typically had one pre-ART visit, one baseline CD4 test and perhaps a creatinine clearance test (see Tables 3 and 4), for a cost of $14.50.

Using the results from Table 4, for example, the cost per patient month on ART after initiation can also be estimated. For example, after subtracting the $14.50 cost of pre-ART care from the total costs, the cost of ART-patient care per month of ART (30 days per month) after initiating is about $19 for patients in either group. Given the daily cost of $0.32 for ARV medications (national first line fixed dose combination), the monthly cost just for medications is $9.60 and then about $10 per month for other resources (clinic visits and laboratory tests).

As presented in the cost-outcomes literature, mean costs (from Table 3) and the proportion achieving the primary outcome (Table 1) can be used to estimate the cost to achieve one successful outcome [15]. Using the results from Tables 1 and 3, the cost to achieving one successful outcome is $134.66/0.64 = $210 for the early-FTIC group and $130.13/0.60 = $217 for the AGL-FTIC group. However, we conclude that this cost-per-successful-outcome metric is not especially useful in this context because it assumes that all care provided to patients not achieving the outcome provided no benefit to them. For example, from Table 5, these patients did receive on average about 3 months of ART over the study follow up period.

While outcomes related to treatment, such as timing of initiation, retention, viral suppression could easily vary by patient sex or age (the two characteristics available to the study), little reason exists to expect that costs for adults newly initiated on ART would vary with basic patient characteristics such as age or sex, given that patients in both study groups were adults, relatively healthy (e.g., no tuberculosis or cryptococcal meningitis), not pregnant, and well balanced on baseline characteristics, as all such patients would be expected to receive the same services.

This analysis is subject to limitations and challenges. First, as noted in the methods section, we used routinely collected data extracted mainly from TIER.Net to evaluate provider costs for patient care during the early roll out of the adherence guidelines. Other than a short period at the start of the study when the AGL evaluation study team worked with the facility staff on data enhancement and completion of missing data, the overall quality of the data collected and entered into TIER.Net at the 24 study sites was largely beyond the control of the AGL study.

Second, this analysis assumes that the unit cost for a pre-ART visit is similar to the costs of a post-initiation follow up visit. However, in the study clinics during the study follow up period, most patients in each model of care received about one pre-ART visit (i.e., in Tables 3 and 4), consequently, whatever unit cost is used, the cost for pre-ART visits would be the same for each model of care and would have minor effects on total costs. While there is no evidence that cost of a pre-ART visit with the new AGL guidelines involved substantially more staff time for patient counseling, with one pre-ART visit in both study groups (from Tables 3 and 4), any additional cost for a pre-ART visit for the AGL-FTIC group would translate directly into the same additional cost for total care and treatment. For example, after adjusting for inflation to 2017, the cost of an adherence counseling session was estimated at $1.31 as part of a rapid initiation study [7]. Adding such minor additional costs for adherence counseling into this analysis would have no meaningful effect on costs.

In addition, the unit cost for a clinic visit and ART initiation visit are based on studies completed in Gauteng Province, but both were completed in public health clinics where staff are paid based on governmental salary scales (and a link to the government’s Department of Public Service and Administration salary scales in ***S1 File***). The basic unit cost for a follow up visit of $7.61 is equivalent to 30 minutes for a middle grade public health nurse.

Third, during the study period, adherence counseling services provided to patients were poorly documented by the NDOH and clinics. Since clinics did not always include this information in patient records and, if they did, the information recorded rarely documented the content of those sessions, the AGL evaluation study could not determine the number of adherence counseling sessions actually received by patients in either model of care and the fidelity to implementing FTIC according to the national guidelines. However, qualitative research from focus group discussions suggested that relatively few of the focus group participants in either model of care actually received adherence counseling **after initiation** [3].

Finally, upfront costs to provide FTIC services as envisioned in the adherence guidelines, particularly staff training, are not included here. As long as possible training of trainers and training of clinic staff are modest in scale and the trainees continue working and using their new skills, training costs amortized over a few years and across many patient interactions would have minor effects on visit costs. For budgetary purposes, such information would be perhaps useful, along with the minor supplies envisioned in the adherence guidelines but not included here, such as a flip chart for presenting information or creating a hard copy of an adherence plan (a few pages maximum).

## Conclusion

By 2016, which was four years after the NDOH recommendation of fast-track ART initiation for some categories of patients, South African patients receiving standard of care typically only had one clinic visit before ART initiation. By the time that national adherence guidelines that included FTIC were rolled out, there existed little room to reduce the number of pre-ART clinic visits [21–24]. The FTIC component of the overall AGL evaluation reported relatively similar initiation and retention outcomes between the two models of care, and this companion cost-outcomes study found similar costs of HIV care and treatment for patients in each model of care for patients achieving the primary outcome (Table 3).

Despite the revision and clarification around the timing and content of the counselling sessions for fast-track initiation counselling, the ability to measure or monitor fidelity to implementation was limited for both models of care included in this analysis. Improved record systems that document the timing and content of counseling services provided to patients will facilitate future assessments of these national guidelines and help to determine more accurately the role these counselling sessions play in facilitating initiation and retention in care and adherence to treatment in the months after initiation.

## Supporting information

S1 File Patient characteristics and unit costs. (XLSX)

S2 File (DOCX)

S3 File (PDF)
