# Supplementary material for: Fast-track treatment initiation counselling in South Africa: A cost-outcomes analysis
Source: PLoS One. 2021 Mar 18;16(3):e0248551. doi: 10.1371/journal.pone.0248551 (PMC7971492; doi:10.1371/journal.pone.0248551)
Supplement: S3 File — (PDF) [file pone.0248551.s003.pdf]

Completed by Bruce Larson. 11 March 2019

Section/item	Item No	Recommendation	Reported on page No/ line No
**Title and abstract**			
Title	1	Identify the study as an economic evaluation or use more specific terms such as “cost-effectiveness analysis”, and describe the interventions compared.	Yes (cost outcomes)
Abstract	2	Provide a structured summary of objectives, perspective, setting, methods (including study design and inputs), results (including base case and uncertainty analyses), and conclusions.	Yes (see Abstract)
**Introduction**			
Background and objectives	3	Provide an explicit statement of the broader context for the study.	Line 70 -89
Present the study question and its relevance for health policy or practice decisions.			Line 98-132
**Methods**			
Target population and subgroups	4	Describe characteristics of the base case population and subgroups analysed, including why they were chosen.	Line 96-102 and referenced AGL study protocol
Setting and location	5	State relevant aspects of the system(s) in which the decision(s) need(s) to be made.	Line 98-132 and referenced AGL study protocol
Study perspective	6	Describe the perspective of the study and relate this to the costs being evaluated.	Line 91-94
Comparators	7	Describe the interventions or strategies being compared and state why they were chosen.	Line 96-132 and referenced AGL study protocol
Time horizon	8	State the time horizon(s) over which costs and consequences are being evaluated and say why appropriate.	Line 158-164 and referenced AGL study protocol
Discount rate	9	Report the choice of discount rate(s) used for costs and outcomes and say why appropriate.	Not needed (9 month follow up, only variable inputs)
Choice of health outcomes	10	Describe what outcomes were used as the measure(s) of benefit in the evaluation and their relevance for the type of analysis performed.	Line 224-246
Measurement of effectiveness	11a	*Single study-based estimates:* Describe fully the design features of the single effectiveness study and why the single study was a sufficient source of clinical effectiveness data.	Line 136-140, Line 76-84, referenced AGL study protocol, and referenced Pascoe JAIDS outcomes manuscript
	11b	*Synthesis-based estimates:* Describe fully the methods used for identification of included studies and synthesis of clinical effectiveness data.	Not relevant
Measurement and valuation of preference based outcomes	12	If applicable, describe the population and methods used to elicit preferences for outcomes.	Not relevant
Estimating resources and costs	13a	*Single study-based economic evaluation:* Describe approaches used to estimate resource use associated with the alternative interventions. Describe primary or secondary research methods for valuing each resource item in terms of its unit cost. Describe any adjustments made to approximate to opportunity costs.	Line 166-216 and supplemental file 1
	13b	*Model-based economic evaluation:* Describe approaches and data sources used to estimate resource use associated with model health states. Describe primary or secondary research methods for valuing each resource item in terms of its unit cost. Describe any adjustments made to approximate to opportunity costs.	Not relevant
Currency, price date, and conversion	14	Report the dates of the estimated resource quantities and unit costs. Describe methods for adjusting estimated unit costs to the year of reported costs if necessary. Describe methods for converting costs into a common currency base and the exchange rate.	Line 189-192 and supplemental file 1
Choice of model	15	Describe and give reasons for the specific type of decision-analytical model used. Providing a figure to show model structure is strongly recommended.	Not relevant
Assumptions	16	Describe all structural or other assumptions underpinning the decision-analytical model.	Not relevant
Analytical methods	17	Describe all analytical methods supporting the evaluation. This could include methods for dealing with skewed, missing, or censored data; extrapolation methods; methods for pooling data; approaches to validate or make adjustments (such as half cycle corrections) to a model; and methods for handling population heterogeneity and uncertainty.	Line 135-246 and supplemental file 1
**Results**			

Study parameters	18	Report the values, ranges, references, and, if used, probability distributions for all parameters. Report reasons or sources for distributions used to represent uncertainty where appropriate. Providing a table to show the input values is strongly recommended.	Not relevant
Incremental costs and outcomes	19	For each intervention, report mean values for the main categories of estimated costs and outcomes of interest, as well as mean differences between the comparator groups. If applicable, report incremental cost-effectiveness ratios.	Not relevant for cost outcomes (consequences) evaluation
Characterising uncertainty	20a	*Single study-based economic evaluation:* Describe the effects of sampling uncertainty for the estimated incremental cost and incremental effectiveness parameters, together with the impact of methodological assumptions (such as discount rate, study perspective).	Not relevant
	20b	*Model-based economic evaluation:* Describe the effects on the results of uncertainty for all input parameters, and uncertainty related to the structure of the model and assumptions.	Not relevant
Characterising heterogeneity	21	If applicable, report differences in costs, outcomes, or cost-effectiveness that can be explained by variations between subgroups of patients with different baseline characteristics or other observed variability in effects that are not reducible by more information.	Results stratified by outcome (see Table 4 and 5)
**Discussion**			
Study findings, limitations, generalisability, and current knowledge	22	Summarise key study findings and describe how they support the conclusions reached. Discuss limitations and the generalisability of the findings and how the findings fit with current knowledge.	377-447
**Other**			
Source of funding	23	Describe how the study was funded and the role of the funder in the identification, design, conduct, and reporting of the analysis. Describe other non-monetary sources of support.	Line 565-570
Conflicts of interest	24	Describe any potential for conflict of interest of study contributors in accordance with journal policy. In the absence of a journal policy, we recommend authors comply with International Committee of Medical Journal Editors recommendations.	Line 549
